# Spermatorrhœa

**Published:** 1874-05

**Authors:** 


*Spermatorrhœa.*—

℞.—Ferri sulph.,

Acid. nitrici . . . . . aa. ʒj.

Aquæ, . . . . . . . ℥j.

Quiniæ sulph, . . . . . . ʒj.

Acid. arsenios.

Strychniæ sulph. . . . . . aa. gr. j.

M.—S. Gtt. xx., t. i. d.

The above prescription is regarded as very efficacious.

